# Barriers to universal health coverage in Republic of Moldova: a policy analysis of formal and informal out-of-pocket payments

**DOI:** 10.1186/s12913-015-0984-z

**Published:** 2015-08-11

**Authors:** Taryn Vian, Frank G. Feeley, Silviu Domente, Ala Negruta, Andrei Matei, Jarno Habicht

**Affiliations:** Department of Global Health, Boston University School of Public Health, 801 Massachusetts Avenue, Crosstown Building 3rd floor, Boston, MA 02118 USA; World Health Organization Country Office in Republic of Moldova, Sfatul Tarii Str. 29, MD-2012 Chisinau, Republic of Moldova; National Bureau of Statistics, 106 Grenoble Str., MD-2019 Chisinau, Republic of Moldova

**Keywords:** Eastern Europe, Health expenditures, Health policy, Health systems, Out-of-pocket payments, Informal payments, Republic of Moldova, Universal health coverage

## Abstract

**Background:**

Universal Health Coverage seeks to assure that everyone can obtain the health services they need without financial hardship. Countries which rely heavily on out-of-pocket (OOP) payments, including informal payments (IP), to finance total health expenditures are not likely to achieve universal coverage. The Republic of Moldova is committed to promoting universal coverage, reducing inequities, and expanding financial protection. To achieve these goals, the country must reduce the proportion of total health expenditures paid by households. This study documents the extent of OOP payments and IP in Moldova, analyses trends over time, and identifies factors which may be driving these payments.

**Methods:**

The study includes analysis of household budget survey data and previous research and policy documents. The team also conducted a review of administrative law intended to control OOP payments and IPs. Focus groups, interviews, and a policy dialogue with key stakeholders were held to validate and discuss findings.

**Results:**

OOP payments account for 45 % of total health expenditures. Sixteen percent of outpatients and 30 % of inpatients reporting that they made OOP payments when seeking care at a health facility in 2012, more than two-thirds of whom also reported paying for medicines at a pharmacy. Among those who paid anything, 36 % of outpatients and 82 % of inpatients reported paying informally, with the proportion increasing over time for inpatient care. Although many patients consider these payments to be gifts, around one-third of IPs appear to be forced, posing a threat to health care access. Patients perceive that payments are driven by the limited list of reimbursable medicines, a desire to receive better treatment, and fear or extortion. Providers suggested irrational prescribing and ordering of tests as drivers. Providers may believe that IPs are gifts and do not cause harm for patients and the health system in general.

**Conclusions:**

Efforts to expand financial protection should focus on reducing household spending on medicines and hospital-based IPs. Reforms should consider ways to reduce medicine prices and promote rational use, strengthen administrative controls, and increase incentives for quality health care provision.

**Electronic supplementary material:**

The online version of this article (doi:10.1186/s12913-015-0984-z) contains supplementary material, which is available to authorized users.

## Background

The goal of Universal Health Coverage (UHC) is to assure that all people have access to the health services they need, of sufficient quality to be effective, and that people do not suffer financial hardship in paying for health care services [1]. Countries which rely heavily on out-of-pocket (OOP) payments to finance total health expenditures are not likely to achieve UHC, as formal user fees and informal payments can cause financial hardship and push households into poverty [2–5]. Reliance on OOP payments also can result in greater inequality in use of services based on socio-economic status [6–9].

Financial protection is a problem in many of the Former Soviet Union (FSU) countries, with households spending a larger and less predictable portion of total resources on health compared with EU-15 countries [6]. Household OOP spending on health in EU-15 countries accounts for 18 % of total health expenditures on average [6], whereas OOP spending accounted for over 28 % of total health spending in seven out of eight FSU countries analyzed [10]. Informal payments (IPs) are especially problematic in Central Asia and Eastern Europe [11–14]. In Bulgaria, 13 % of outpatients and 33 % of inpatients reported paying informally for treatment [15], while 26–29 % of Albanians were “suggested to make” a payment for services that should have been provided free of charge in government facilities, signifying these were likely forced payments and not gifts [16].

The Republic of Moldova is one of the countries which has struggled with high OOP payments, estimated at 44.9 % in 2011 [17], and with public concern over IPs [18, 19]. With a population of 3.5 million and GDP per capita of $2,038 (2012, data do not include the districts on the left side of the river Dniester and the municipality of Bender), Moldova is one of the poorest countries in Europe although it has experienced economic growth in recent years [20, 21].

In approaching health reforms to achieve UHC, Moldova has taken what the World Health Organization calls a “whole systems approach” [4] seeking not only to create and expand health insurance enrollment, but also to adopt health policies which would increase equity in service use, improve efficiency and expand financial protection for all citizens [22–25]. The mandatory health insurance system was created by law in 1998 and became operational in 2004 [26]. Administered by the National Health Insurance Company (CNAM), the program provides access to an essential package of emergency, primary, and inpatient services without charge [18]. The package of services includes drugs in inpatient settings and a limited list of reimbursable medicines for outpatient care [19, 26]. For a majority of conditions, family doctors serve a gatekeeping function, and referral is required to access secondary and tertiary services.

An amendment to the Law on Mandatory Health Insurance in 2009 ensured that families living below the poverty line, even if formally self-employed, would automatically receive fully subsidized health insurance [26, 27]. Additional amendments in 2010 provided all citizens, regardless of income level, with access to free primary health care services provided by family doctors [24] and pre-hospital emergency care services [19]. To implement this reform, the health insurance system allocated additional funding with a focus on improving rural access. About 30 % of the insurance program’s budget was spent on primary care including prescription medicines in 2012 [28]. Funding has strengthened the family medicine model and enhanced autonomous management of primary care centers, independent of hospitals, so they are able to separately contract with CNAM [29]. Coverage under the mandatory health insurance system was 79.7 % in 2011; however, patients still often pay in the private sector for uncovered services such as some high technology diagnostic tests, medicines not on the Reimbursed Drugs List, and for secondary/tertiary services if they are not referred [18].

Targets set in CNAM’s institutional strategy propose that by 2017 the insurance program will decrease the share of OOP payments as a proportion of total health expenditures to 36 %, decrease the share of expenditures for medicines as a proportion of OOP payments to 65 % while at the same time expanding the Reimbursed Drugs List, and decrease the proportion of patients making IPs at primary and outpatient care to 20 % and at hospitals to 45 % (among those who paid anything for care). CNAM plans to achieve these targets through the expansion of health insurance coverage and benefits (as mentioned above), and other strategies in the early stages of implementation, including assuring protection of the rights of insured people through better customer service, complaint management, and a beneficiary protection system, and further increasing access and quality of care by reducing waiting time to obtain services, conducting medical audits, and using innovative performance-based financing mechanisms for contracting with hospitals [23].

At a broader level, health sector reforms since 2012 have tried to reduce OOP payments by gradually increasing salaries of health workers [30], increasing budget for covered prescription medicines, introducing mandatory use of international non-proprietary names (INN) in prescribing, and implementing a medicines pricing policy reform whereby the manufacturer’s price is set at the time of product registration using external reference price information [31]. Debates in recent years have also recognized corruption as a key development challenge, and the need to increase transparency as a cross-sectoral issue [32].

Evidence suggests that reforms may be working. For example, use of services is increasing: by 2010 people in Moldova had 6.5 outpatient contacts per person per year, a number which exceeded the European Union average of 6.3 contacts [26]. In addition, Moldova compares favorably with other FSU countries on access: a study in 2010 found that 70 % of respondents saw a doctor when they felt they needed to, more than in Armenia, Azerbaijan, Belarus, Georgia, Kazakhstan, Russia or Ukraine [10]. But the specific impact of reforms on OOP payment and IP trends is not well understood.

The objective of this study is to document trends in OOP payments and IPs in Moldova, looking especially at how the rate of OOP payments may vary by insurance status and socio-economic status. We use multiple sources of data, including desk review of policies and documents, stakeholder feedback, analysis of annual Household Budget Survey data and the ad hoc module on health administered every two years, secondary analysis of an inpatient survey of OOP payments and IPs, legal review, and the findings from a policy workshop. The study discusses differences in perceptions about the factors driving formal payments and the largely hidden practice of IPs, and makes recommendations to reduce OOP payments and IPs taking into account values, institutions, and capacities of Moldova’s health sector.

## Methods

In order to address this complex topic we used methodology triangulation, blending and integrating multiple sources of data to increase the credibility and provide evidence of the validity of our analysis [33]. The methods included a review of policy documents, a legal review, and analysis of quantitative household care-seeking and expenditure data. In addition, interviews and focus groups were conducted to share findings of the analysis with stakeholders and to incorporate their reactions.

The study was carried out between July 2013 and January 2014 and included three missions in-country and data analysis. The work was guided by the World Health Organization (WHO) Country Office in Republic of Moldova, whose staff conceived of the study and participated in data collection. Researchers from the Boston University School of Public Health collaborated on the study design, implementation, data analysis and writing. STROBE scientific reporting guidelines were followed as appropriate to a policy analysis of this nature (Additional file 1).

### Desk review and initial interviews

The study began with a review of policy documents and data from Moldova and other countries in the region to develop a preliminary analytical framework. This framework was presented during an in-country mission in August 2013 in consultation with representatives of the WHO Country Office in Republic of Moldova. Interviews were conducted with 24 officials from the Ministry of Health, CNAM, the National Bureau of Statistics (NBS), the Moldova National Anti-Corruption Centre, development partners, civil society organizations, and health facilities. The analytical framework was then revised based on the results.

### Stakeholder feedback

A second visit was conducted in October 2013 to gather feedback from stakeholders on the analytical framework and possible policy options. We conducted 7 focus group discussions (FGD), 3 with providers and administrators, and 4 with patients at two large (>500 bed) hospitals and a Family Medicine Centre in the Chisinau area, and one district hospital (<450 beds). A total of 5 administrators and 24 providers (23 doctors and 1 nurse) participated in the provider focus groups, and 17 patients or care givers participated in the patient focus groups. Convenience sampling was used to gather participants. Sample size was limited by the time available, but we believe that thematically, saturation was reached. All participants were told that their participation was voluntary and findings would be anonymous. No one who was approached refused to participate. Patient focus groups were 2–6 patients each. The FGD questions explained that the government was concerned about high OOP payments, and asked questions about motivation for payments people make for care; advantages and disadvantages of the payments for the providers and patients; and ideas for reform. We also analyzed qualitative data reported from a 2012 study of barriers to care in Moldova for additional insights [27]. That study was based on analysis of six focus group discussions with a total of 50 citizens, and 16 in-depth interviews (IDI) with government managers and public sector social service providers.

### Legal review

Based on our desk review, and a similar study of health insurance law and IPs conducted in Albania [34], we identified the most pertinent administrative law governing the insurance reimbursement and patient payment processes, including English translations of the Law on Compulsory Health Insurance (No 1585—XIII, 27 Feb 1998) as amended through 2012 as well as the Government Decision No 1636 Regarding the Approval of the Type Contract for Health Care Provision within the Compulsory Health Insurance, 18 December 2002. We also considered the main insurer and provider relations influencing OOP payments and considered provisions related to enforcement of policies not to force additional payments from patients while services are covered from public funds.

### Quantitative data

We reviewed two sources of data on care seeking behaviors and OOP payments for health care services. The first is a Household Budget Survey (HBS) conducted by the NBS each month, with data summarized by year. The nationally representative survey interviews members of 9,768 randomly selected households each year, stratified by residential area and statistical zones. Respondents were informed on confidentiality of data and gave written informed consent. The response rate was 63.7 % in 2009, 60.5 % in 2010, 66.2 % in 2011, and 60.4 % in 2012. Across the four years, reasons for non-response were diverse, but the most important reasons were that the survey was not considered important or respondents lacked time to fill out questionnaire (about 48 % of non-respondents on average), or no one at home after three attempts (30 % of non-respondents) [35]. The section of the survey which is about health status and health expenditure asks about care seeking from providers with a recall period of 4 weeks for outpatient care and 12 months for inpatient care. Respondents are queried on OOP payments related to outpatient care (primary care doctors, specialist doctors, and diagnostic testing centers), inpatient care (hospitalization experience), dental care, and medicines purchased through pharmacies (not including medicines received free of charge during hospital stay). We examined data from 2009 to 2012 to calculate the percent of patients who paid a formal or informal payment at the primary care level, hospital level, or for medicines purchased as part of their care in a health facility. The data exclude dental care and people who only sought care at a pharmacy for self-treatment. The data allow us to categorize patients as insured versus uninsured, and to analyze results by consumption quintile (1 = poorest, 5 = least poor) as a measure of socio-economic status. The analysis weighted survey data to represent the national population [35].

The second data source was an ad hoc module on health which is collected as part of the HBS. This specific ad hoc module is implemented every other year, rather than annually. This module provides data on reasons for not seeking care when ill. Data were analyzed for 2008, 2010, and 2012. Sample size for the ad hoc module was 2,442 households (3,760 respondents) in 2008, 2,442 households (3,444 respondents) in 2010, and 1,348 households (including 3,424 respondents) in 2012 [36, 37]. We had approval from the National Bureau of Statistics of the Republic of Moldova to use the HBS and Ad Hoc Module data.

In addition to the HBS data, we performed a secondary analysis of a survey conducted in 2011 by the Center for Health Policies and Studies (PAS), an independent non-governmental organization (NGO) in Moldova. The PAS report was translated into English from Romanian by an experienced professional translator. The study approached 5,600 households to select 1,204 people who had been hospitalized in the past 12 months. This study provides a larger sample of hospitalized patients than the NBS HBS. Finally, we reviewed data on medical staff salaries from 2006 to 2013 obtained from the Ministry of Health, to compare with perceptions of low salaries as a driver of IPs.

### Policy workshop

A policy workshop was held at the Ministry of Health in Chisinau on January 31, 2014 to present the study findings and discuss the policy options. Participants included the Minister of Health, Vice Ministers, heads of services, facility directors, other government agency representatives, a representative of the Doctors’ League, and civil society organizations. The insights of workshop participants were incorporated into the results.

### Analysis

Using the various data sources, we analyzed trends in total health expenditure, and OOP payments as a proportion of total health expenditures, for Moldova compared to other countries; trends in proportion of people who experienced a health problem but did not seek care due to financial reasons; trends in the percentage of people who made an OOP payment for services received at outpatient facilities or as an inpatient; and trends in the percentage of people who paid informally (of those who paid anything at all). We analyzed how the rate of OOP payment varied by insurance status (insured versus uninsured) and by socio-economic status (consumption quintile). For qualitative data we used inductive thematic analysis to examine collated data from in-depth interviews and focus group discussions to identify patterns of meaning and to define and name themes [38].

### Definitions

**Fig. 1:**
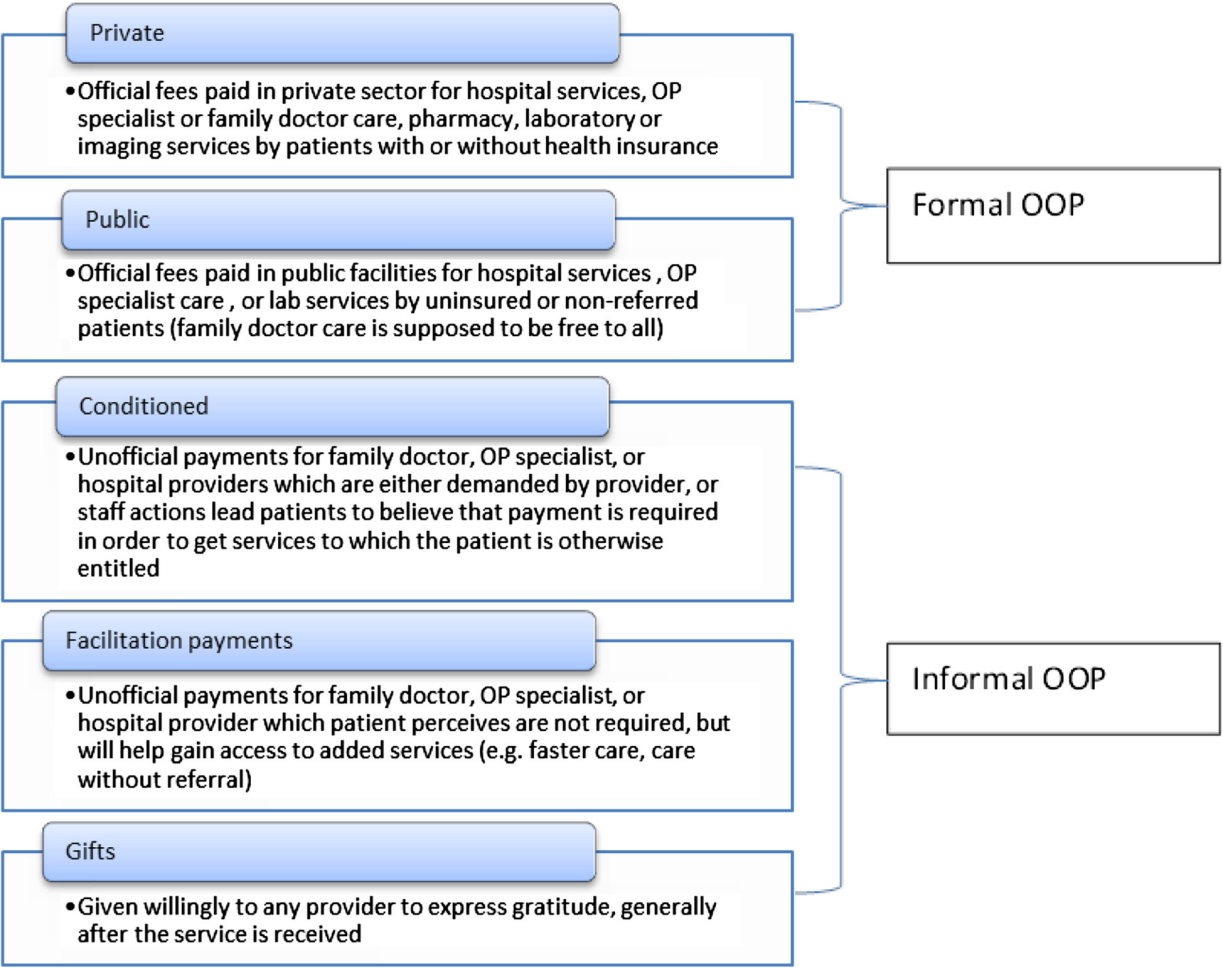
Types of formal and informal OOP payments in the Republic of Moldova

OOP payments are defined by the World Bank as “any direct outlay by households, including gratuities and in-kind payments, to health practitioners and suppliers of pharmaceuticals, therapeutic appliances, and other goods and services whose primary intent” is to enhance or restore health status [39]. This includes official user fees at public facilities as well as fees paid in the private sector. IP is defined as “a direct contribution, which is made in addition to any contribution determined by the terms of entitlement, in cash or in-kind, by patients or others acting on their behalf, to health care providers for services that the patients are entitled to” [40]. During stakeholder feedback interviews and focus groups, we found that IPs are sometimes referred to locally in three categories: conditioned payments (perceived by patients as necessary in order to receive services), facilitation payments (offered voluntarily by patients to obtain something outside the basic service package entitlement), and gifts (given freely to express gratitude). See Fig. 1.

### Ethics statement

Terms of reference for the study were approved by the Ministry of Health of the Republic of Moldova, and the WHO Country Office as a policy analysis. Informed consent was obtained from stakeholders participating in interviews and discussions. No individual level patient data were collected.

## Results

### Trends in total health expenditure

Total health expenditure as a proportion of GDP was 11.7 % in 2012, a 75 % increase since 2000 (Table 1). This share of spending on health is equal to Switzerland and Canada, and well above the 9.6 % average for OECD countries [41]. Yet in absolute terms, total spending on health is only 490 $US PPP per capita [17], and Moldova ranked 48th in the WHO European region on this measure [26]. Moreover, the high proportion of spending on health in relation to GDP is driven by private spending, which is least supportive of UHC goals: private health expenditures comprised 54.5 % of total health expenditure in Moldova in 2012, an increase of 5.8 % from 2000 (Table 1). OOP payments by private households account for 83.2 % of private spending; other sources of private expenditure on health include 15.8 % by non-profit institutions serving households, e.g. NGOs (defined by WHO as institutions which are not predominantly financed and controlled by government, that provide goods or services to households free or at prices that are not economically significant) and 1 % spending on private health insurance/other [17]. Thus, private households’ OOP payment on health as a proportion of total health expenditures in Moldova is 45.3 %. This is higher than many other FSU countries such as Belarus, Kazakhstan, Russia and Ukraine, but lower than Armenia, Azerbaijan, and Georgia [10]. By comparison, in EU-15 countries OOP spending was 18 % of total health expenditures [6].

**Table 1:** Trends in health expenditure 2000–2012 selected years, Republic of Moldova

	2000	2006	2007	2008	2009	2010	2011	2012	% Change 2000–2012
Total Health Expenditure (THE) as % GDP	6.7	10.6	10.9	11.4	12.5	11.7	11.4	11.7	74.6 %
General government expenditure on health (GGHE) as % THE	48.5	44.4	45.2	47.2	48.5	45.8	45.5	45.5	−6.2 %
Private expenditure on health as % THE	51.5	55.6	54.8	52.8	51.5	54.2	54.5	54.5	5.8 %
Private household OOP payment as % of private expenditure on health	83.3	82.9	83.3	85.4	84.8	82.8	82.6	83.2	−0.8 %
Private household OOP payment as % of THE	42.9	46.1	45.6	45.1	43.7	44.9	44.9	45.3	5.7 %

Source: WHO, 2013 [17]

Data from the HBS ad hoc module on health show that the proportion of people who said they did not seek care when they needed it due to financial reasons fell by half, from 29.2 % in 2008 to 14.8 % in 2012 (Table 2). Among those who had not sought care when they felt they needed it in 2012, 29.1 % in the poorest quintile said they could not afford either services or drugs. Table 2 also highlights that progress has not been equal in urban versus rural areas: between 2008 and 2012, in urban areas there was 70.3 % reduction in the percentage of the population who did not seek care for financial reasons, compared with only 38.7 % reduction among people living in rural areas.

**Table 2:** People who experienced a health problem but did not seek care due to financial reasons (2008–2012, selected years)

Characteristic	2008	2010	2012	% change 2008–2012
Overall	29.2	20.9	14.8	−49.3 %
Residence				
Urban	20.9	11.3	6.2	−70.3 %
Rural	36.4	28.3	22.3	−38.7 %
Income Quintile				
1	43.6	40.4	29.1	−33.3 %
2	35.4	28.3	24.2	−31.6 %
3	28.0	20.1	16.6	−40.7 %
4	28.3	11.2	7.1	−74.9 %
5	13.4	11.5	5.0	−62.7 %
Insurance Status				
Insured	26.5	17.4	12.7	−52.1 %
Not insured	37.6	28.9	20.6	−45.2 %

Source: National Bureau of Statistics, Household Budget Survey ad hoc module on health, collected every other year

### Portion of patients who made OOP payment

**Fig. 2:**
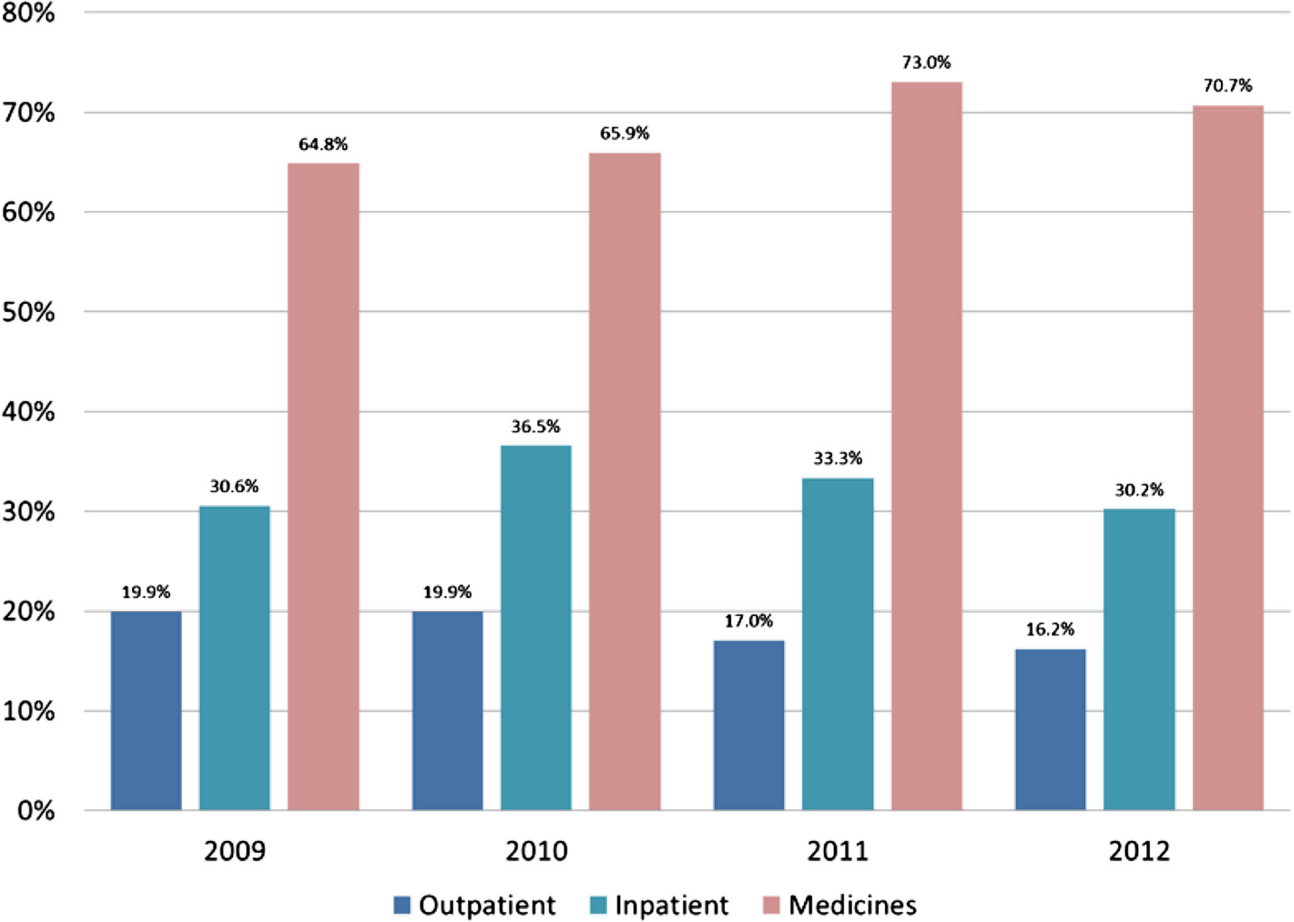
Percent of patients making OOP payment when seeking care in the last 4 weeks, 2009–2012

Figure 2 shows that the percentage of people making OOP payment at outpatient facilities has decreased from 19.9 % in 2009 to 16.2 % in 2012. The rate of OOP payment at inpatient facilities rose from 30.6 % in 2009 to 36.5 % in 2010, but then also started to decline to 30.2 % in 2012. Data from the PAS hospitalized patient survey showed a somewhat lower rate of formal OOP payment in hospitals, but a high rate of any hospital-based OOP payment if we count both formal and informal payments. The PAS study found that 22.2 % of inpatients had made formal payments for services provided (defined as payments made at the cash desk of the inpatient unit), while 37.9 % made an IP directly to a health worker. If there was no overlap, the combined percentage of patients making any OOP payment would be 60.1 %; however, there is likely overlap between these categories. The NBS survey data show that the percentage of people who sought care with a provider and also paid for medicines at a pharmacy increased 9 % from 64.8 % in 2009 to 70.7 % in 2012.

### Proportion of patients who paid informally

**Fig. 3:**
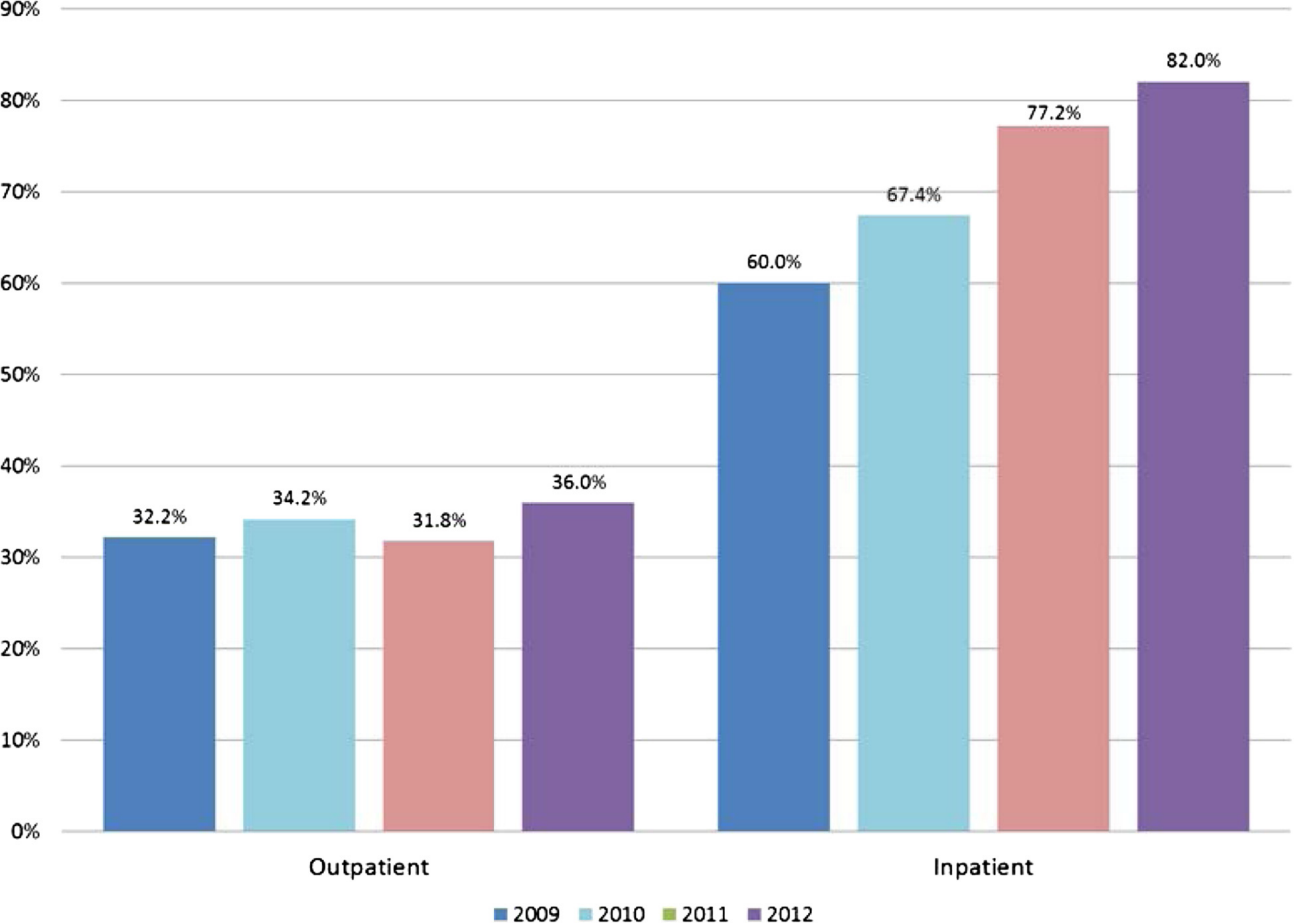
Percent of patients making an informal payment in the last 4 weeks, of those who made any OOP payment, 2009–2012

Figure 3 shows that the rate of informal payment in outpatient settings is fairly steady: of those who made any OOP payment for care, about 32 % of patients in 2009 reported making an informal payment when seeking outpatient care at a health facility, compared to 36 % of patients in 2012. However, the rate of informal payment for inpatient care shows a steep increase over time, from 60 % of patients in 2009 to 82 % of patients in 2012.

Data from the PAS study provide additional detail on the types of payments. Of those who made an IP in a hospital, 61.6 % said they had given willingly (i.e. a gift such as money or a present), 23.2 % said the payment was imposed by a health worker (i.e. a conditioned payment), and 14.7 % said they had given both a gift and a conditioned payment. Combining categories would suggest that over three-quarters of patients had made a gift, and over one-third of inpatients surveyed had been obliged to make an informal payment for services to which they were entitled.

Taking into account that the majority of people in Moldova do not report making an OOP payment when seeking care, the combined probabilities are lower: overall, only 5.8 % of all outpatients and 24.6 % of inpatients reported making an informal payment.

### Rates of OOP payment and insurance status

**Fig. 4:**
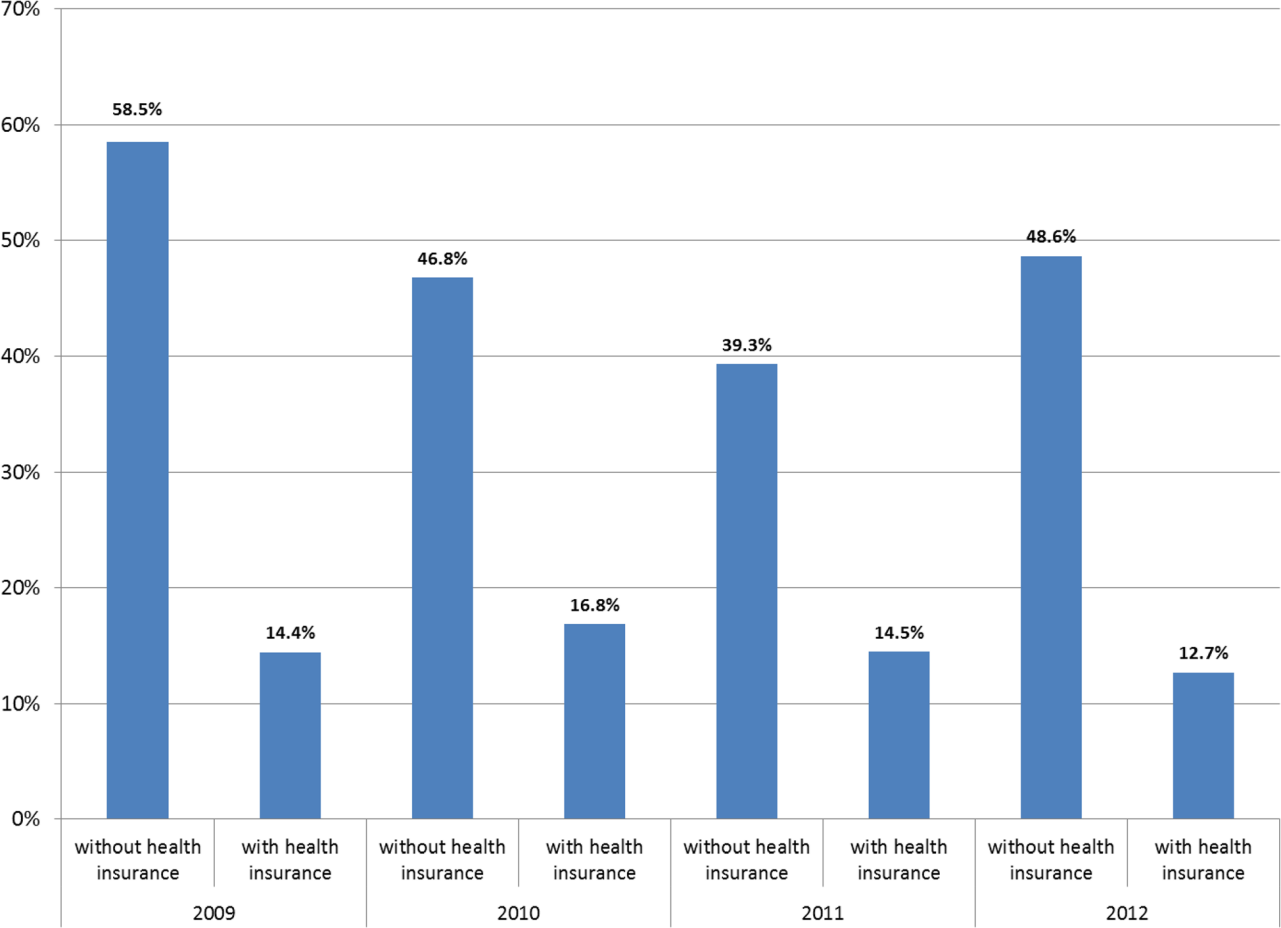
Percentage of uninsured and insured outpatients making an OOP payment when seeking care in last four weeks, 2009–2012

**Fig. 5:**
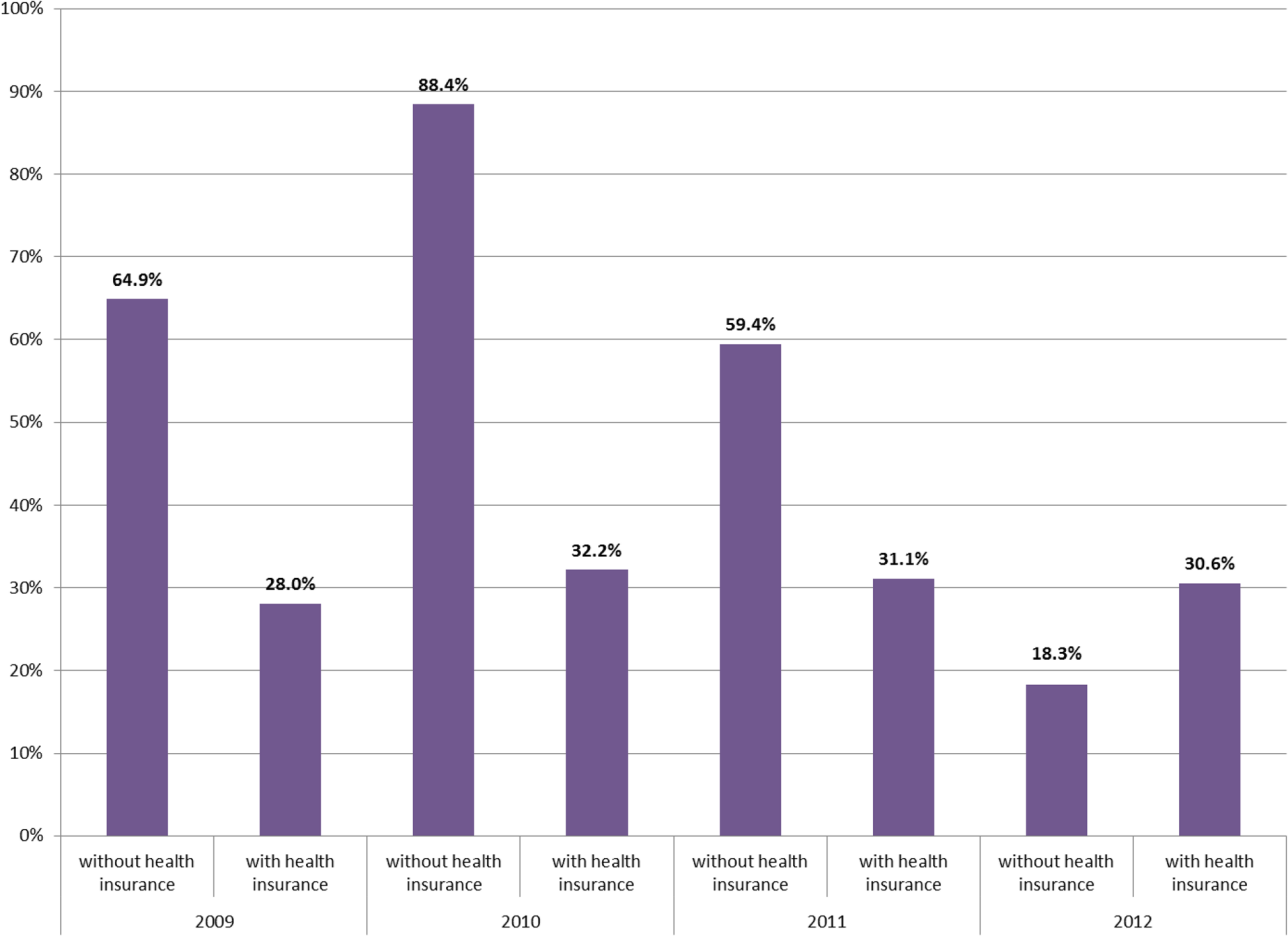
Percentage of uninsured and insured inpatients making an OOP payment when seeking care in last four weeks, 2009–2012

For outpatient OOP payment, uninsured were 3.8 times more likely to make an OOP payment in 2012 compared to insured patients (49 % vs. 13 %) (Fig. 4). However, the data for inpatient care are less clear (Fig. 5). While in 2011, uninsured inpatients were almost twice as likely to make an OOP payment as insured patients (59 % vs. 31 %), in 2012, insured patients were more likely to make an OOP payment (31 % of those with health insurance made an OOP payment, compared to 18 % of those without insurance).

### Rates of OOP payment and socio-economic status

Analysis of OOP payment by socio-economic status shows that the poor are less likely to make facility-based OOP payment when seeking outpatient care (Table 3). In 2012, less than 10 % of patients in consumption quintile 1 (poorest) made an OOP payment for outpatient facility-based care, compared to 29 % in quintile 5 (least poor). However, for inpatient care we see no obvious trend. In 2012, the proportion of inpatients making an OOP payment was virtually the same in quintile 1 (36 %) and quintile 5 (35 %). For medicines, over 77 % of patients in the poorest quintile who sought facility-based care also purchased medicines in 2012, compared to only 57 % of patients in quintile 5.

**Table 3:** Percent of patients who sought facility-based care and made an OOP payment, by consumption quintile (1 = most poor, 5 = least poor) and type of care, 2009–2012

Year/Consumption Quintile	Outpatient	Inpatient	Medicines
2009			
Quintile 1 (poorest)	7.4 %	26.6 %	70.9 %
Quintile 2	12.9 %	17.3 %	68.8 %
Quintile 3	16.1 %	32.1 %	71.2 %
Quintile 4	18.6 %	37.5 %	69.3 %
Quintile 5 (least poor)	32.0 %	30.7 %	52.4 %
2010			
Quintile 1 (poorest)	9.2 %	31.2 %	65.0 %
Quintile 2	11.8 %	20.9 %	76.1 %
Quintile 3	13.5 %	32.5 %	71.7 %
Quintile 4	20.2 %	53.5 %	65.6 %
Quintile 5 (least poor)	31.9 %	33.0 %	57.2 %
2011			
Quintile 1 (poorest)	6.9 %	27.2 %	80.0 %
Quintile 2	8.0 %	34.0 %	82.3 %
Quintile 3	10.2 %	20.4 %	81.7 %
Quintile 4	19.9 %	40.3 %	72.5 %
Quintile 5 (least poor)	28.6 %	37.5 %	58.6 %
2012			
Quintile 1 (poorest)	9.5 %	36.2 %	77.5 %
Quintile 2	8.7 %	31.5 %	77.9 %
Quintile 3	12.8 %	11.1 %	76.9 %
Quintile 4	13.0 %	30.4 %	71.9 %
Quintile 5 (least poor)	29.2 %	35.0 %	57.4 %

Source: National Bureau of Statistics, Household Budget Survey (annual)

### OOP payment amounts

According to NBS data, patients who sought and reported paying for outpatient care in 2012 paid an average of 231 MDL or €15 Euro for services (median 120 MDL, €8). Those who reported also paying for medicines paid an average of 299 MDL or €19 Euro (median 191 MDL, €12). Patients who reported paying for inpatient care said they paid 1,253 MDL or €81 Euro (median 600 MDL, €39). The recall period was 4 weeks for outpatient payment estimates and 12 months for inpatient estimates; data were not collected on spending on inpatient care plus medicines for this time period. Compared to the average salary in Moldova (3,550 MDL or €231 Euro per month [30]), this means that people who pay for care are spending 7–8 % of monthly income on outpatient episodes, and up to 35 % of monthly income on inpatient care.

Of those inpatients who reported paying a direct payment in the PAS study, the average amount paid in 2010 was 1,449 MDL or €90 (median 700 MDL or €44). The recall period was one year. Those who underwent surgical interventions were 70 % more likely to pay than all other patients, while those with higher incomes tended to pay more formally than those with lower incomes. Among the 38 % of patients who made an IP, the average amount paid was 1,193 MDL or €74 (median 400 MDL or €25).

### Perceptions of patients and providers

Drivers of formal OOP payment identified by patients relate to the limited list of reimbursed drugs, a desire for services not covered by insurance (such as acupuncture) and a perception that care is better in the private sector. Stakeholders also mentioned that patients seek care in the private sector to avoid waiting lines for procedures in public facilities. Providers mentioned that these waiting lines are imposed because of ceilings on the number of procedures which will be reimbursed by the insurance program due to limited budget. Some stakeholders said patients are able to consult with a doctor without paying, but OOP payment for medicines and diagnostic tests were harder to avoid. Drivers for IPs mentioned by patient stakeholders included the desire for faster care (avoid waiting list), fear that quality would be affected if you did not pay, and gratitude for a good health outcome such as the birth of a child. Some patients described conditioned payments required for surgical care. Table 4 provides illustrative quotes.

**Table 4:** Drivers of formal and informal payments

Formal payment	Informal payment
Medicine is not covered by insurance	Desire for faster care
*The hospital gives some medicines, but [a patient] might need an expensive one the hospital doesn’t have.* – Patient FGD 2013	*I said ‘I can pay.’ The physician is happy and I am happy. And I did not stay in line.* –IDI 2012, insured male
*If you want your child to be healthy and get well, you need to pay a lot for medicines.* – Patient FGD 2013	*I leave some money on the table. If you go downstairs to the payment office, when you come back the doctor is already busy and you have to wait. It is easier to pay him/her directly.* –IDI 2012, uninsured male
Sense that inexpensive medicines in public sector must not be good quality, and that “better” medicines are offered in private sector	Fear of poor quality:
*How can a drug be good if it costs 10 times less than another medicine?* – Provider FGD 2013	*When I was hospitalized, there were some people telling us we shouldn’t pay since we had health insurance…But I was paying anyway to assure myself that everything would be fine. I was very worried and I just wanted to know that everything would be OK.* –IDI 2012, insured female
*If you don’t want the hospital’s drugs, but you want your own drugs, then you have to purchase them.* – Patient FGD 2013	*The relatives pay the staff to tend their patient since there aren’t enough staff and the staff can’t take care of all the patients. The staff aren’t asking for these payments, but the relatives are paying.* – Patient FGD 2013
Service not covered by insurance	
*The acupuncture treatment …is expensive and not covered by insurance.* – Patient FGD 2013	*Unless you put some cash in their pockets, they won’t even look at you…they don’t even come close to you and don’t even check on you…I noticed an immediate change after we paid. They looked after [my wife] and came regularly to check on her. The workers from there expected to be paid.* –IDI 2012, insured male
Uninsured or not referred	Wish to thank providers
*[People prefer] to pay for a hospitalization and medicines rather than purchasing insurance.* – Patient FGD 2013	*For the birth of my two children, I gave money. Nobody forced me. I gave 5000 or 6000 lei (317–380 euro) with all my heart.* – IDI 2012, insured male
*If I wanted to see the specialist right away, I would have had to pay.* – Patient FGD 2013	*I think it is normal, not forced.* – Patient FGD 2013
	*About informal payments, I think it is something about individuals giving flowers and candies. No one is imposing this.* – Patient FGD 2013

Source: Stakeholder feedback (focus groups and in-depth interviews) collected in October 2013; qualitative data from previous research (WHO, 2012 [27])

Providers mentioned several reasons for formal OOP payment, including high tech diagnostic tests which public hospitals are not able to perform or which are rationed due to cost (so patients end up seeking care in the private sector); patients who need a second or third line medication not covered by insurance, or who prefer a different medicine from the one on the Reimbursed Drugs List; and the health needs of patients who have not purchased insurance or did not obtain a required referral. Providers believed most IPs were gifts, which they described as token presents (candy, flowers) or small amounts of cash given after discharge. Most providers thought that gift giving is a strong cultural value and does not cause harm. When asked about the underlying causes of OOP payment, providers mentioned low reimbursement rates from the insurance fund to facilities; ceilings on the number of cases which the insurance fund will reimburse; low salaries; over-utilization of services (too many referrals for testing, over-prescribing by doctors); drug advertising and inappropriate self-treatment by patients; poor quality medicines; and patient attitudes toward gift giving.

Doctors felt beleaguered by anti-corruption awareness campaigns which they thought inaccurately portrayed doctors as corrupt. They believed the press and household survey reports were exaggerating the problem of IPs, and that this was negatively affecting society’s view of the medical profession. “The image of the doctor is being destroyed because of the image created by the government through these surveys,” one doctor said. He warned that if the medical profession is tarnished, people will begin to leave the profession creating a doctor shortage. Doctors also raised the issue that focusing on bribes during the patient-provider interaction is a way of diverting attention from wrong-doing by senior health officials, i.e. grand corruption which could involve much higher sums of money or have more deleterious effects.

## Discussion

### OOP payment mainly driven by medicines

Our study sought to analyze the pervasiveness of OOP payments and IPs in Moldova and to understand trends and causes of these payments. The findings suggest that health reforms to improve financial protection are working in Moldova: the proportion of people who did not seek care for financial reasons decreased between 2008 and 2012 from 29.2 to 14.8 %. Yet, access for the very poor is still a problem, as 29.1 % of the poorest quintile said they could not afford services or drugs in 2012. In addition, the rate of OOP payments is still high at 44.9 % of total health expenditures, largely due to spending on medicines not covered by insurance [10]. Sixteen percent of outpatients and 30 % of inpatients made an OOP payment for medical services within a health facility in 2012. People who pay for care are spending 7–8 % of monthly income on outpatient episodes, and up to 35 % of monthly income on inpatient care.

Over 70 % of patients who sought facility-based care ended up paying for medicines in 2012, an increase of 9 % since 2009. This suggests that while the policy to extend free coverage for primary care services has reduced the likelihood of making OOP payment at outpatient facilities, access to medicines still requires an ability to pay. The finding that poorer patients may pay more frequently for medicines than less poor (77 % vs. 57 %) is difficult to explain, and requires more research to understand how rates of payment may be correlated with other demographic or systems-level factors.

Payments for medicines are driven by the limited scope of the Reimbursed Drugs List, prescribing practices which may shift patients to medicines which are not reimbursed, the practice of prescribing and using too many medicines in treatment (“polypharmacy”), and the high unit prices of medicines [42]. Although all drugs dispensed at hospitals are 100 % covered for patients with insurance, most of the medicines prescribed in outpatient settings are only reimbursed partially, i.e. 50 %, 70 %, 90 %. This means either patients have to make an OOP payment in a private pharmacy, or some have suggested that patients try to get hospitalized so the drugs will be free [27]. Some doctors in public practice are reportedly prescribing medicines which are not covered by insurance, possibly due to lack of confidence in or knowledge about standard treatment guidelines, or doubts about the quality of medicines on the Reimbursed Drugs List. These doctors may suggest that a different (generally more expensive and/or brand name) medicine is better. Since laws also permit doctors to have ownership stake in pharmacies, prescribing patterns may be influenced by financial benefit to the doctor-owner, or there could be a kickback relationship between prescriber and pharmacist.

Finally, polypharmacy may be driving up OOP payment through unnecessary use of medicines. A study which examined prescriptions written in 2011 found that 7.8 % of cases showed evidence of over-prescribing by physicians [42]. In addition, despite a reform to require doctors to prescribe using INN for medicines in 2012, some doctors still prescribe using brand names, and generic substitution by pharmacists is not allowed [42]. This means that the prices paid by patients for prescribed medicines are higher than necessary. Patient attitudes may also be contributing to polypharmacy. For convenience or because they do not believe doctors have considered all possible treatment options, patients may seek medicines at private pharmacies or public pharmacies in primary health care units without first consulting with a clinician. Sometimes antibiotics and injectable medicines are sold to patients without prescription [43].

Polypharmacy and self-treatment can have negative consequences for health, in addition to increasing OOP payment: it is harder for patients to adhere fully to instructions when they are taking multiple medicines, and patients may take incomplete or insufficient treatments (possibly fueling antimicrobial resistance) or suffer side effects or drug interactions [44]. Polypharmacy and irrational prescribing are exacerbated by inadequate supervision of prescribing and lack of monitoring by government, civil society, and professional bodies [42].

Medicine unit prices are high for a variety of reasons, i.e. procurement agents with limited skills in price negotiation, restrictive intellectual property rules, and the country’s small size which limits bargaining power [45]. For formal OOP payment, specific interventions which could help control the cost of medicines include stronger quality control measures, greater transparency in pharmaceutical regulation, continuous training of doctors, controls to reduce influence by pharmaceutical representatives, and educational programs for patients [42, 45]. Depending on the public budget available, the government could also consider adding more medicines to the Reimbursed Drugs List. The latter strategy would not contain total health expenditures, but it would shift more of the burden of payment from OOP payment to government.

### Informal payments declining: drivers and perceptions are mixed

Our findings suggest that IP rates may be declining over time. In a 2007 study, 48 % of people in Moldova reported that IPs were sometimes or always needed to access health care services [46]. In contrast, in our study only 5.8 % of outpatients and 24.6 % of inpatients reported making IPs when seeking care. These data are lower than patterns documented in the Transparency International (TI) Corruption Barometer 2013 survey, which reported 38 % of people in Moldova said they had had to pay something informally to access care. Discrepancies may be due to differences in survey methods (the TI study uses a 12 month recall period for all respondents, while the NBS survey uses a one month recall period for outpatients and 12 months for inpatients; in addition TI survey respondents were asked to report on behavior of anyone living in the household, not just their own behavior). NBS data confirm that IPs are mainly a problem in hospitals, while the PAS study provides evidence that patients most frequently think of these payments as gifts.

The drivers of IPs in Moldova—fear that people may not be able to get care of good quality otherwise, desire for faster care, and an expression of gratitude—are similar to reasons documented in Hungary [47, 48], Albania [49, 50], and other countries [11]. Researchers in Hungary found that people who have accepting attitudes toward IPs are more likely to think IPs are expressions of gratitude, and to believe that services are underfunded and doctors are not well paid [51]. Some providers in Moldova did suggest that low salaries are driving IPs, and salary data support this assumption to an extent. We found that the annualized physician salary in Moldova including 15 % vacancy pay is 8,774 US$PPP, or about 1.4 times the average wage rate in the country.^1^ A study comparing remuneration of general practitioners to the average wage in 14 OECD countries found that remuneration of GPs varies from twice the average wage in Finland and Czech Republic to 3.5 times greater than average wage in the United States and Iceland, so in comparison with this benchmark salaries in Moldova are on the low side. Unlike other FSU countries, Moldova does not have a problem of excess physicians having to share a limited health budget for personnel: the country had 313 doctors per 100,000 population in 2009, compared to 330 per 100,000 for the WHO European Region [26]. However, other studies suggest that raising wages alone is not enough to deter corrupt practices such as bribe seeking [52], and that efforts should try to link pay to performance [53, 54].

Attitudes toward the payments may influence the type of IP: for example, in Turkey being grateful or following customs is a reason for in-kind gifts (50 %), but is rarely a driver for cash IPs [55]. Further analysis of Moldova data in the future might explore such differences.

### Protecting patient interests through incentive systems, enforcement, and information

Compulsory IPs are thought to be more deleterious to health outcomes, both because they deter the poor from accessing services, but also because they affect resource allocation in such a way that services are not used by those who would benefit most [11, 56]. Although the rate of IP in Moldova appears low and motivated in large part by gratitude or customs, policy makers should still be worried about the one-third of payments in hospitals that are not gifts, and the fact that financial access is still a problem for the very poor. To control IPs, policy makers should consider strengthening CNAM contractual provisions to ban IPs and to deduct payment from providers whose employees continue to accept them. The Health Insurance Law could be amended to specify that demand for payments other than those authorized under the insurance program is an attempt to violate the rights of the insured and would incur defined penalties. Members of the insurance program could be provided with information on covered services and medicines, permitted copayments, percentage of the cost of medicines covered, and generic medicine equivalents. Such information is available now in the main hall of medical institutions and in some pharmacies, but the information is difficult to find and not presented in an easily digested form. Some work is needed to improve methods and instruments for communication of information to insurance members in ways that target vulnerable populations and truly increase patient understanding. Insured patients should also know how to access and feel comfortable using grievance redress mechanisms, an important anti-corruption strategy [57, 58]. Other strategies to consider include increasing individual incentives for providers through revised payment structures emphasizing performance-based payment [59, 60], and incentivizing organizations by awarding grants to facilities to support innovative strategies to increase transparency, as has been done in Mongolia [61] and Vietnam [62].

### Limitations

This study has several limitations. First, because of the way data were collected in the HBS, it does not include people who only sought care at a pharmacy for self-treatment (an estimated 31.4 % of patients self-treat at pharmacies or alternative care providers). As such, our figures may under-estimate the total proportion of people making any OOP for medicines. This is potentially important especially as prescriptions are not fully enforced and many medicines are available in the pharmacies without prescription allowing self-treatment. In addition, due to problems in quality of HBS data we were not able to analyze household expenditures for dental care. The majority of dental care is paid directly through OOP payment and only emergency dental care is covered by the public insurance program. Additional analysis should be conducted to understand the role of dental care as a driver of OOP payment.

A second limitation of the study is that we had limited data sources for monitoring IP practice and could not measure how these payments may vary with socio-economic status and with insurance status. Future studies should try to measure changes in the practice of making IPs of various types (e.g. facilitation payments, gifts, compulsory payments) and correlate IPs with other demographic factors. Our qualitative interviews and focus groups also were limited in geographic scope due to budget constraints; however, the analysis raised themes which were similar to those documented previously in Moldova, suggesting that the issues we identified are persistent concerns. Finally, our legal analysis was limited to laws which were translated into English. Although the most important laws and amendments were available in English, it is possible that our legal review omitted documents which describe payment control procedures and exist only in the Romanian language.

## Conclusion

UHC embodies the goal of effective health coverage; that is, to increase the probability that individuals will receive the health interventions that they need with appropriate quality, and that the costs involved do not erode financial protection. Health reforms adopted by the government of Moldova have helped to improve access to care on average from 2008 to 2012, and the rate of primary care consultations per capita is high. During past few years important stakeholders including government leaders and citizens, have become concerned about OOP payment and this topic has reached the policy agenda of the health authorities and beyond. However, factors documented in our research suggest that there may still be unmet needs in Moldova and that financial protection is not yet assured. Continuing issues include the limited list of reimbursed medicines (which results in patients purchasing medicines OOP), failure of government to motivate generic prescribing and rational use of medicines, perceptions of patients and providers that better medicines and higher quality healthcare services are available in the private sector, and continued compulsory informal payments for some services in the hospital sector. Moreover, data suggest there are differences in access to services by place of residence, income and insurance status which are a cause for concern. On the positive side, there is broad recognition of the need to strengthen governance and reduce expectations for informal payment in all sectors and throughout government. The Ministry of Health and CNAM need to continue working with other stakeholders in the country to strengthen transparency and accountability with particular focus on the supply side including health care providers. This can be complemented with expansion of insurance coverage to move forward on the path to universal health coverage while targeting also equitable access to care.

## Additional file

Additional file 1:
**STROBE Statement—Checklist of items that should be included in reports of** ***cross-sectional studies*** **MS1678068119131527 Barriers to Universal Coverage in Republic of Moldova: a policy analysis of formal and informal out-of-pocket payments.** (DOC 93 kb)

## Abbreviations

CNAM: National Health Insurance Company
EU: European Union
FGD: Focus group discussion
FSU: Former Soviet Union
GDP: Gross domestic product
HBS: Household Budget Survey
IDI: In-depth interview
IP: Informal payments
MDL: Moldovan Leu
NBS: National Bureau of Statistics
NGO: Non-governmental Organization
OECD: Organization for Economic Cooperation and Development
OOP: Out-of-pocket
PAS: Center for Health Policies and Studies
PPP: Purchase power parity
TI: Transparency International
UHC: Universal health coverage
WHO: World Health Organization

## Footnotes

1 Often doctors can occupy more than one vacancy and their monthly salary will increase accordingly. According to statistics, on average they occupy (accumulate) up to 1.25 vacancies. Exchange and conversion rates obtained from Ministry of Health as of October 2013.
