# Supplementary material for: Barriers to universal health coverage in Republic of Moldova: a policy analysis of formal and informal out-of-pocket payments
Source: BMC Health Serv Res. 2015 Aug 11;15:319. doi: 10.1186/s12913-015-0984-z (PMC4531477; doi:10.1186/s12913-015-0984-z)
Supplement: Additional file 1: — STROBE Statement—Checklist of items that should be included in reports of cross-sectional studies MS1678068119131527 Barriers to Universal Coverage in Republic of Moldova: a policy analysis of formal and informal out-of-pocket payments. (DOC 93 kb) [file 12913_2015_984_MOESM1_ESM.doc]

**STROBE Statement**—Checklist of items that should be included in reports of ***cross-sectional studies***

**MS1678068119131527** Barriers to Universal Coverage in Republic of Moldova: a policy analysis of formal and informal out-of-pocket payments

|  | **Item** | **Recommendation** | **Comment** |
| --- | --- | --- | --- |
| **Title and abstract** | 1 | (a) Indicate the study’s design with a commonly used term in the title or the abstract | Title and abstract mentions that the design was a policy analysis. Abstract describes using several methods and data sources, including analysis of household budget survey data, review of previous research and policy documents, review of administrative law, focus groups, interviews (p. 2) |
|  |  | (b) Provide in the abstract an informative and balanced summary of what was done and what was found | Abstract includes background, methods, results, and conclusion and is balanced (p. 2-3) |
| **Introduction** |  |  |  |
| Background/rationale | 2 | Explain the scientific background and rationale for the investigation being reported | Background on Moldova and the problem of out-of-pocket (OOP) payments are discussed in the context of universal coverage goals (p. 4-6). |
| Objectives | 3 | State specific objectives, including any pre-specified hypotheses | Objective is stated on p. 6. We are documenting trends in OOP payments and informal payments (IPs), and how rates of payment may vary by insurance status and socio-economic status. The analysis also looks at drivers of payments. |
| **Methods** |  |  |  |
| Study design | 4 | Present key elements of study design early in the paper | The policy analysis design and methods of data collection are described in detail on pages 7-10. |
| Setting | 5 | Describe the setting, locations, and relevant dates, including periods of recruitment, exposure, follow-up, and data collection | The dates of primary data collection (August 2013, October 2013, January 2014) are described and related to the types of data collected and locations (p. 7-9). |
| Participants | 6 | (a) Give the eligibility criteria, and the sources and methods of selection of participants | Selection of stakeholders for gathering feedback is described on p. 7-8. Convenience sampling was used to gather data from patients and providers at several different types of facilities. |
| Variables | 7 | Clearly define all outcomes, exposures, predictors, potential confounders, and effect modifiers. Give diagnostic criteria, if applicable | The outcome of interest from Household Budget Survey is clearly described as proportion of people paying. Recall period differs for outpatient and inpatient and is explained. Data are separately tabulated based on insurance status and socioeconomic status as explained on p. 8-9. |
| Data sources/ measurement | 8* | For each variable of interest, give sources of data and details of methods of assessment (measurement). Describe comparability of assessment methods if there is more than one group | See p. 7-10 for full description. |
| Bias | 9 | Describe any efforts to address potential sources of bias | On p. 20-21, we discuss limitations of the study and how this may affect generalizability. One limitation of study is that we could not gather data on spending by people who only sought care in pharmacy. Another is that qualitative data are only from a few facilities. Finally, health laws were viewed in translation only. |
| Study size | 10 | Explain how the study size was arrived at | See p. 7-8 where we mention the number of qualitative interviews/focus groups conducted and participants in the focus groups. Sample size was limited because of budget, but we believe saturation was reached. |
| Quantitative variables | 11 | Explain how quantitative variables were handled in the analyses. If applicable, describe which groupings were chosen and why | See p. 8-9 for full description. It made sense to separate outpatient from inpatient utilization, and to examine people with insurance coverage separately from those without. It made sense to compare OOP payment rates for those in different consumption quintiles. |
| Statistical methods | 12 | (a) Describe all statistical methods, including those used to control for confounding | We calculated proportions (proportion making OOP payment when seeking care, and proportion making informal payment out of those who paid anything at all). See p. 8-10. |
|  |  | (b) Describe any methods used to examine subgroups and interactions | As mentioned we separately examined patients based on insurance status and income quintile as defined in the household budget survey questions. We did not examine interactions. |
|  |  | (c) Explain how missing data were addressed | NA |
|  |  | (d) If applicable, describe analytical methods taking account of sampling strategy | NA |
|  |  | (e) Describe any sensitivity analyses | NA |
| **Results** |  |  |  |
| Participants | 13 | (a) Report numbers of individuals at each stage of study—eg numbers potentially eligible, examined for eligibility, confirmed eligible, included in the study, completing follow-up, and analysed | 29 administrators and providers, and 17 patients participated in focus groups. Household budget survey is collected from 9,768 households (p.7). Ad hoc module on health data collected from 1,348 to 2,442 households, depending on the year. |
|  |  | (b) Give reasons for non-participation at each stage | For focus groups, no one who was approached refused to participate (p. 7). For HBS survey, response rate was 60.4% (p. 8). There were many reasons for non-response, but the main reasons included thinking survey was unimportant or not having time (48% of non-respondents), or no one at home (30%). |
|  |  | (c) Consider use of a flow diagram | NA |
| Descriptive data | 14 | (a) Give characteristics of study participants (eg demographic, clinical, social) and information on exposures and potential confounders | NA |
|  |  | (b) Indicate number of participants with missing data for each variable of interest | NA |
| Outcome data | 15* | Report numbers of outcome events or summary measures | We report proportion of patients who made payments, see Table 2 and 3, and Fig. 2-3 |
| Main results | 16 | (a) Give unadjusted estimates and, if applicable, confounder-adjusted estimates and their precision (eg, 95% confidence interval). Make clear which confounders were adjusted for and why they were included | Not relevant to this study |
|  |  | (b) Report category boundaries when continuous variables were categorized | Not relevant to this study |
|  |  | (c) If relevant, consider translating estimates of relative risk into absolute risk for a meaningful time period | NA |
| Other analyses | 17 | Report other analyses done—eg analyses of subgroups and interactions, and sensitivity analyses | The mixed method study included analysis of rates of payment and insurance status (p. 13); rates of payment and socio-economic status (P. 13); average payment amounts (p. 14); perceptions of stakeholders (p. 14-15) |
| **Discussion** |  |  |  |
| Key results | 18 | Summarise key results with reference to study objectives | Key results are summarized by themes, i.e. OOP payment rates driven by medicines (p. 16, para. 1-2) and declining IPs and mixed drivers (p. 18-19). Recommendations for protecting patient interests are also discussed p. 19-20. |
| Limitations | 19 | Discuss limitations of the study, taking into account sources of potential bias or imprecision. Discuss both direction and magnitude of any potential bias | These are discussed on p. 20-21, including how the limitation may influence results. |
| Interpretation | 20 | Give a cautious overall interpretation of results considering objectives, limitations, multiplicity of analyses, results from similar studies, and other relevant evidence | The study triangulated the problem of OOP payments and informal payments through use of multiple sources of data and by analyzing trends. See p. 20-21. |
| Generalisability | 21 | Discuss the generalisability (external validity) of the study results | See discussion (p. 16-19) and limitations sections (p. 20). |
| **Other information** |  |  |  |
| Funding | 22 | Give the source of funding and the role of the funders for the present study and, if applicable, for the original study on which the present article is based | Mentioned on p. 22. |
